# Muscarinic Long-Term Enhancement of Tonic and Phasic GABA_A_ Inhibition in Rat CA1 Pyramidal Neurons

**DOI:** 10.3389/fncel.2016.00244

**Published:** 2016-10-26

**Authors:** Soledad Domínguez, David Fernández de Sevilla, Washington Buño

**Affiliations:** ^1^Instituto Cajal – Consejo Superior de Investigaciones CientificasMadrid, Spain; ^2^Centre National de la Recherche Scientifique, Paris Descartes University, UMR 8118, ParisFrance; ^3^Departamento de Anatomía, Histología y Neurociencia, Facultad de Medicina, Universidad Autonoma de MadridMadrid, Spain

**Keywords:** tonic current, ambient GABA, extrasynaptic receptors, synaptic receptors, inhibitory feedback

## Abstract

Acetylcholine (ACh) regulates network operation in the hippocampus by controlling excitation and inhibition in rat CA1 pyramidal neurons (PCs), the latter through gamma-aminobutyric acid type-A receptors (GABA**_A_**Rs). Although, the enhancing effects of ACh on GABA**_A_**Rs have been reported (Dominguez et al., 2014, 2015), its role in regulating tonic GABA_A_ inhibition has not been explored in depth. Therefore, we aimed at determining the effects of the activation of ACh receptors on responses mediated by synaptic and extrasynaptic GABA_A_Rs. Here, we show that under blockade of ionotropic glutamate receptors ACh, acting through muscarinic type 1 receptors, paired with post-synaptic depolarization induced a long-term enhancement of tonic GABA**_A_** currents (***t***GABA**_A_**) and puff-evoked GABA**_A_** currents (***p***GABA_A_). ACh combined with depolarization also potentiated IPSCs (i.e., phasic inhibition) in the same PCs, without signs of interactions of synaptic responses with ***p***GABA_A_ and ***t***GABA_A_, suggesting the contribution of two different GABA_A_ receptor pools. The long-term enhancement of GABA_A_ currents and IPSCs reduced the excitability of PCs, possibly regulating plasticity and learning in behaving animals.

## Introduction

Acetylcholine (ACh) plays a fundamental role in the regulation of network operation in the hippocampus (Watanabe et al., 2006; Connelly et al., 2013; Dominguez et al., 2014, 2015). CA1 pyramidal cells (PCs) participate in circuits involved in cognition and spatial navigation, however, the underlying cellular mechanism by which ACh acts on CA1 networks have been insufficiently explored. In PCs ACh can induce a long-term potentiation (LTP) of excitatory synapses through post-synaptic mechanisms (Markram and Segal, 1990; Fernandez de Sevilla et al., 2008; Fernandez de Sevilla and Buno, 2010; Dennis et al., 2015). ACh can control inhibitory synapses in PCs both through presynaptic (Wu and Saggau, 1997; Alger, 2002; Kano et al., 2009) and post-synaptic mechanisms (Kittler and Moss, 2003; Bannai et al., 2009; Castillo et al., 2011; Luscher et al., 2011; Dominguez et al., 2014, 2015). In addition, *via* the control of network activity in the hippocampus ACh can regulate learning and memory (Hasselmo, 2006; Robinson et al., 2011). GABA_A_Rs elicit the tonic current (***t***GABA**_A_**), which hyperpolarizes CA1 PCs, reduces network excitability (Semyanov et al., 2003; Semyanov et al., 2004), and regulates information processing (Mitchell and Silver, 2003; Chadderton et al., 2004) and behavior (Belelli et al., 2009; Brickley and Mody, 2012). We have reported that a long-term enhancement of IPSCs induced by ACh combined with depolarization (ACh+depolarization) was paralleled by a potentiation of tonic inhibition in PCs (Dominguez et al., 2015), but the LTP of tonic inhibition remains insufficiently explored. Therefore, the central aim of this work was to determine the effects of the activation of cholinergic receptors and membrane depolarization on responses resulting from the activation of synaptic and extrasynaptic GABA**_A_**Rs.

Here, we report that in immature rats, under blockade of glutamatergic ionotropic receptors, a brief pulse of ACh on the apical dendritic shaft while the PC was repeatedly depolarized during the experiment caused a durable increase in tonic GABA**_A_** current (***t***GABA**_A_**), and a LTP of puff-evoked GABA**_A_** currents (***p***GABA_A_), that we call ***p***LTPextra. These effects were matched by a LTP of IPSCs that we have termed GABA**_A_**-LTP (Dominguez et al., 2014, 2015). The parallel long-term enhancement of tonic and phasic inhibition caused a strong reduction of the excitability of PCs that possibly regulates network operation, plasticity and learning in behaving animals. The enhancement of both tonic and phasic inhibition followed similar time course and rules. However, we could not observe changes in IPSCs following GABA puff, suggesting that: (*i*) the long-term boost of tonic and phasic inhibition shared key mechanisms; (*ii*) GABA puffs and GABA released by inhibitory interneurons activated different GABA_A_R pools; (*iii*) GABA “spillover” did not play an important role in the effects of ACh+depolarization on synaptic responses.

## Materials and Methods

### Ethical Approval and Animal Handling

Procedures of animal care and slice preparation approved by the CSIC followed the guidelines laid down by the European Council on the ethical use of animals (Directive 2010/63/EU) and with every effort made to minimize the suffering and number of animals.

### Slice Preparation

Most of the materials and methods used here were reported previously in detail (Dominguez et al., 2014, 2015). Immature Wistar rats (14–20 days old) of either sex, deeply anesthetized with isoflurane, were decapitated and their brain removed and submerged in cold (≈4°C) carbogen-bubbled artificial cerebrospinal fluid (ACSF), which contained in mM: 124.00 NaCl, 2.69 KCl, 1.25 KH_2_PO_4_, 2.00 MgSO_4_, 26.00 NaHCO_3_, 2.00 CaCl_2_, 10.00 glucose, and 0.40 ascorbic acid. Transverse slices (300–400 μM) of the hippocampus were incubated >1 h in ACSF at room temperature of 20–22°C. Slices were transferred to an upright microscope equipped with infrared differential interference contrast video microscopy (DIC) and superfused with carbogen-bubbled ACSF (2 ml/min) at room temperature. Recordings were under blockade of glutamatergic ionotropic transmission with 2-amino-5-phosphonopentanoic acid (D-APV; 50 μM) and 7-nitro-2,3-dioxo-1,4- dihydroquinoxaline-6-carbonitrile (CNQX; 20 μM) to inhibit NMDA and AMPA/Kainate receptors, respectively. Picrotoxin (P_i_TX; 50 μM) a specific GABA**_A_**R antagonist (2*S*)-3-[[(1*S*)-1- (3,4-Dichlorophenyl) ethyl] amino-2- hydroxypropyl] (phenylmethyl) phosphinic acid hydrochloride (CGP55485, 2 μM), a specific GABA_B_R antagonist, pirenzepine (1 μM) a specific M1-mAChR antagonist, and nimodipine (10 μM), a specific L-type voltage-gated Ca^2+^ channel (VGCC) antagonist, were added to the ACSF as needed.

### Electrophysiology

**FIGURE 1:**
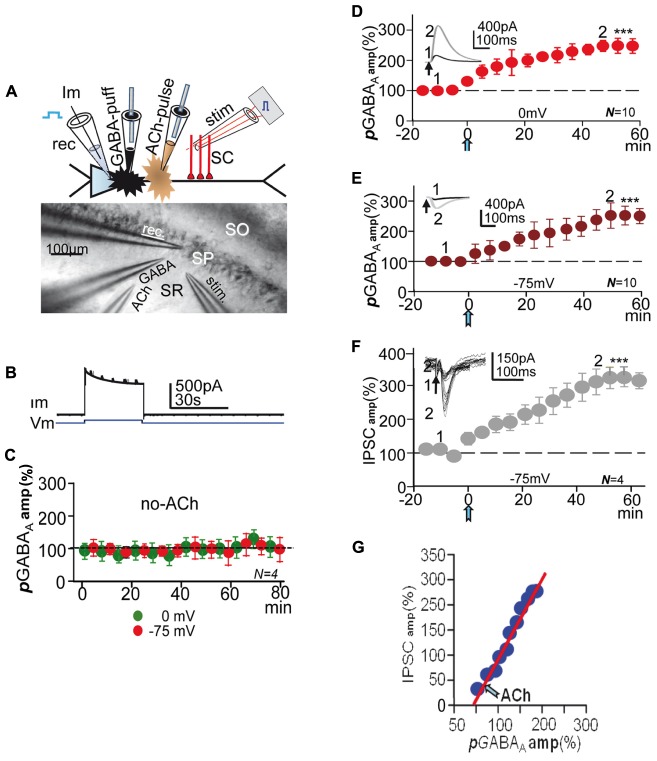
**Experimental setup, controls, and effect of ACh+depolarization.** (**A**, top) Schematic diagram showing the CA1 PC and placement of recording (**rec**), stimulation (**stim**) **GABA-puff** and **ACh-pulse** pipettes. **(A**, bottom) DIC image of CA1 slice showing *stratum-radiatum* (**SR**) -*pyramidale* (**SP**) -*oriens* (**SO**) and placement of **rec, stim, GABA-puff** and **ACh-pulse** pipettes. **(B)** Representative example of currents (**Im**) elicited under control conditions by the first 75 to 0 mV step (**Vm**) and **GABA puffs. (C)** Amplitude versus time plot where each data point represents the mean peak amplitude of IPSCs recorded at 0 (green circles) and -75 mV (red circles), showing the lack of changes in ***p***GABA_A_ during de- and hyperpolarization. **(D)** Amplitude versus time plot where each data point represents the peak amplitude of ***p***GABA_A_s recorded at 0 mV, showing the ***p***LTPextra induced by ACh (blue arrow, as in all other figures). **(E)** Same as **(D)** but recorded at -75 mV in the same PCs. The insets in **(D,E)** show representative ***p***GABA_A_s taken at time points 1 and 2. **(F)** Amplitude versus time plot where each data point represents the mean peak amplitude of IPSCs recorded at -75 mV; rest as in **(D,E)**. The inset shows representative superimposed IPSCs taken at time points 1 and 2 in **(F)**. All recordings were made in the presence of 2 μM **CGP55485**. **(G)**. plot of the mean peak amplitude ***p***GABA_A_ versus that of IPSCs (taken from **E,F**) showing the linear relationship between both post-ACh responses. In **(C–F)** each data point represent values averaged over 5 min epochs. Asterisks indicate the significance level (^∗∗∗^*p* < 0.001).

Whole-cell voltage- and current-clamp recordings were from the soma of CA1 PCs (**Figure 1A**), using patch pipettes (4–8 MΩ) that contained in mM: 140 K-MeSO_4_, 10 HEPES, 10 KCl, 4 Na-ATP, 0.3 Na-GTP and 0.1 EGTA, buffered to pH 7.2–7.3 with KOH. A -65 mV chloride equilibrium potential was calculated with the intra- and extracellular solutions used. Neurons were only accepted if during the experiment the seal resistance was >1 GΩ, the series resistance (7–14 MΩ) did not change >15%, and the holding current did not exceed 300 pA at -75 mV. The average resting Vm was -70.2 ± 3 mV (*N =* 127). In some experiments BAPTA (20 mM), a fast Ca^2+^ chelator or heparin (5 mg/ml), which inhibits IP_3_Rs were added to the pipette solution.

### Stimulation under Voltage-Clamp

Steps from -75 to 0 mV lasting 30 s were applied every 75 s during the experiment (**Figure 1B**), while GABA (500 μM), diluted in the control ACSF was repeatedly puffed through a fine tipped pipette every 5 or 10 s on the apical dendritic shaft of the patched PC (**Figures 1A,B**). In most experiments, stimulation at the *stratum radiatum* (SR) evoked single or pairs (50–100 ms delay) of inhibitory post-synaptic currents or potentials (IPSCs-IPSPs) either in isolation or 2.5 s after GABA puffs. Responses were recorded both at 0 and -75 mV. We analyzed the voltage-dependence of ***p***GABA_A_ with I/V relationships that involved 500 ms duration 10 mV voltage control steps from -100 to +20 mV, applied every 10 s, combined with a puff of GABA. In all experiments following a 15–20 min control recording after attaining the whole-cell configuration, a single 100–300 ms pulse of ACh was applied by iontophoresis though a pipette loaded with ACh (1 M) dissolved in distilled water. The ACh pulse was aimed at the SR close to the base of the apical dendrite of the patched PC. To avoid spurious release of ACh the pipette was withdrawn. Stimulation and recording continued >1 h after the ACh pulse. ACh effects were essentially identical when the pulse was applied during brief interruptions of the depolarizing protocols (≈3 min) or during the protocols, and did not depend on the Vm or inhibitory activity (Dominguez et al., 2014, 2015). In some cases voltage steps (as above) were applied in the absence of ACh (**Figure 1C**).

### Stimulation under Current-Clamp

To determine modifications in the excitability of PCs we estimated the changes in action potential (AP) firing evoked by 1 s duration depolarizing current pulses applied throughout the experiment every 5–10 s at twice the AP threshold intensity (**Figure 7A**). In another group of experiments to estimate the effects on both the enhanced ***t***GABA_A_ and IPSPs on PC excitability we applied 500 ms duration current pulses every 10 s at twice the AP threshold intensity. Current pulses were coupled with paired-pulse stimulation (50–100 ms delay) of inhibitory inputs at the SR both during AP bursts and silent periods between bursts (**Figure 7E**). In both experimental groups stimulation was transiently interrupted (≈3 min) after a 15–20 min control recording and the ACh pulse was applied (**Figure 7B**). Under current-clamp the AP threshold was derived from the current intensity of a 1 s duration depolarizing current pulse just sufficient to bring the cell to AP generation when the PC was at the resting Vm.

### Data Analysis

Data were analyzed with the pClamp programs (Molecular Devices, Chicago, IL, USA) and Excel (Microsoft, Redmond, WA, USA). Peak amplitudes of ***p***GABA_A_s and IPSCs averaged over 5 min epochs were plotted versus time, expressed as a proportion of the baseline amplitude. Analysis of the spontaneous IPSCs (**_S_**IPCSs) activity was performed with the pClamp software. Cumulative probabilities of amplitude and inter-event intervals of **_S_**IPCSs recorded during ≈5 min in control conditions and ≈5 min during the ***p***LTPextra ≈40 min after the ACh pulse were computed. Statistically significant differences were established using the Kolmogorov–Smirnov test. Under voltage-clamp, shifts in the mean pre-ACh holding current (Ih) provided a measure of changes in ***t***GABA_A_ (Dominguez et al., 2014). Ih shifts were confirmed by the change in mean steady current after blocking GABA_A_Rs with P_i_TX (50 μM). To determine the temporal evolution of the peak amplitude of currents evoked by GABA puffs at all successive steps we averaged puff evoked current during each depolarizing step in six experiments and plotted them versus time, expressed as the proportion (%) of the mean value of puff evoked currents triggered during the first step (**Figures 4A,B** and see **Figure 2** in Dominguez et al., 2015). Statistical analysis was performed using Student’s two tail *t*-test and differences were considered statistically significant at ^∗^*P* < 0.05 level, ^∗∗^*P* < 0.01, and ^∗∗∗^*P* < 0.001. Results are given as mean ± SEM (*N* = numbers of cells) and (*n* = number of averaged responses). There were no gender differences in our experiments.

**FIGURE 2:**
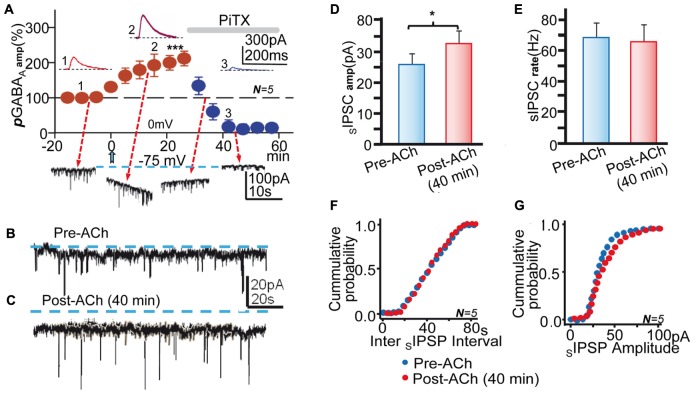
**Blockade of GABA_A_Rs inhibited pLTPextra and tGABA_A_ and reduced the amplitude of _S_IPCS.** (**A**, top) Average ***p***GABA_A_ peak amplitude versus time plot recorded at 0 mV in control ACSF showing the post-ACh ***p***LTPextra and the strong inhibition of ***p***GABA_A_ by P_i_TX (50 μM; horizontal gray bar); rest as in **Figures 1D,E**. The insets show representative ***p***GABA_A_s taken at time points 1, 2, and 3. (**A**, bottom) Representative records showing ***t***GABA_A_ and **_S_**IPSCs recorded at -75 mV taken at time points indicated by arrows. **(B)** Representative pre-ACh spontaneous activity. **(C)** Same as **(B)**, but ≈40 min post-ACh. Note the outward change in holding current (interrupted lines) recorded post-ACh in **(C)**. **(D)** Bar plot showing the Pre-and Post-ACh (≈40 min) mean **_S_**IPSC amplitudes obtained in experiments as in **(B,C)**. **(E)** Same as **(D)**, but showing Pre- and Post-ACh **_S_**IPSC rate. **(F)**. Cumulative probability plots of pre- and post-ACh inter **_S_**IPSC intervals. **(G)** Same as **(F)**, but cumulative probability plots of **_S_**IPSC amplitude. Data in **(D**–**G)** were averaged from **_S_**IPSCs recorded during ≈5 min in control conditions and ≈5 min during the ***p***LTPextra ≈40 min after the ACh pulse. Asterisks indicate the significance level (^∗^*p* < 0.05, ^∗∗∗^*p* < 0.001).

## Results

### In the Absence of ACh the Depolarization Protocol Did Not Modify GABA_A_ Currents

GABA_B_Rs are absent in rat PCs before postnatal day 22 (Nurse and Lacaille, 1999), but there are functional presynaptic GABA_B_Rs in the terminals of CA1 inhibitory interneurons in younger rats (Wu and Saggau, 1997; Dominguez et al., 2015). To avoid the possible activation of presynaptic GABA_B_Rs by the GABA puffed we usually recorded ***p***GABA_A_, ***t***GABA**_A_**, and IPSCs under blockade of GABA**_B_**Rs with CGP55845 (2 μM). Isolated ***p***GABA_A_s had mean peak amplitudes of 575 ± 79 pA at 0 mV and of -294 ± 68 pA at -75 mV (*N* = 10) in the pre-ACh controls (insets in **Figures 1D,E**).

Under voltage-clamp a prolonged presentation of the depolarization protocol (>1 h) in the absence of the ACh pulse did not modify puff-evoked isolated GABA_A_ currents recorded both at 0 and -75 mV (*P* > 0.05; *N* = 4; **Figure 1C**), indicating that repeated depolarization alone was unable to induce long-term changes in extrasynaptic GABA**_A_** currents. We have shown that the same protocol did not modify IPSCs in the absence of ACh (Dominguez et al., 2014).

### ACh+depolarization Induced a Gradual Potentiation of *p*GABA_A_

After the ACh pulse (≈5 min) there was a gradual potentiation of ***p***GABA_A_ or ***p***LTPextra, which in ≈40 min stabilized at mean peak values that were 237 ± 3% of the controls at 0 mV and of 251 ± 4% at -75 mV (*P* < 0.001; *N* = 10; **Figures 1D,E**). Therefore, ***p***LTPextra had similar time-course and reached essentially identical values at 0 and -75 mV (*P* > 0.05 in both cases), suggesting that baseline ***p***GABA_A_ amplitude and the inward or outward Cl^-^ flow did not contribute to the potentiating effects of ACh+depolarization. This was essentially identical to what occurred with IPSCs (Dominguez et al., 2015). An inward current that peaked at ≈30 s and gradually decayed to a steady state in ≈1 min typified the response evoked by the ACh pulse under voltage-clamp at 0 mV (see Figure 1F in Dominguez et al., 2014).

### A Potentiation of IPSCs Accompanied *p*LTPextra

In some experiments, we recorded both IPSCs and ***p***GABA_A_ in the same PCs (see Materials and Methods) under blockade of GABA**_B_**Rs with CGP55845 (2 μM). Stimulation at the SR evoked outward IPSCs (258 ± 11 pA; *N* = 4) at 0 mV and inward IPSCs (-68 ± 8 pA; same cells) at -75 mV in the pre-ACh controls (inset in **Figure 1F**). Following the ACh pulse there was a gradual enhancement of the IPSC recorded at -75 mV that in ≈40 min reached a steady state mean peak value that was 319 ± 6% of the control or GABA_A_-LTP (*P* < 0.01; *N* = 4; **Figure 1F**). Therefore, the synaptic GABA_A_-LTP attained higher steady state values than ***p***LTPextra (127 ± 5%; *P* < 0.05; *N* = 4). A similar IPSC enhancement that reached values of 298 ± 10% of the control value (*P* < 0.01; same sells) was recorded at 0 mV (data not shown, but see Figure 2S in Dominguez et al., 2014).

We also constructed plots of the mean peak ***p***GABA_A_ amplitude versus that of IPSCs to analyze the amplitude relationship between both responses in the same PCs. There was a linear correlation between the mean peak amplitudes of post-ACh ***p***GABA_A_ (abscissa) and IPSCs (ordinates) (slope 1.88; *R* = 0.97; *N* = 4; **Figure 1G**), indicating that IPSC increased more than ***p*GABA_A_**.

### Both *p*LTPextra and the Enhanced *t*GABA_A_ Were Blocked by P_i_TX

To confirm the central contribution of GABA_A_Rs we tested the effects of P_i_TX (50 μM) applied following the ACh pulse when ***p***LTPextra had reached values that were 209 ± 4% of the control (*P* < 0.001; *N* = 5). P_i_TX reduced ***p***GABA_A_ to values that were not significantly different from zero (*P* > 0.05; same cells; **Figure 2A**). These results suggest that an increased response of GABA**_A_**Rs generates ***p***LTPextra. In addition, the magnitude of ***p***LTPextra recorded at 0 mV in control ACSF 25 min after ACh (**Figure 2A**) was essentially identical in control solution and under CGP55845 (**Figure 1D**; *P* > 0.05; *N* = 5 and *N* = 10, respectively), verifying that GABA_B_Rs did not contribute to the enhancing effects of ACh. Moreover, the mean amplitude of control ***p***GABA_A_ was essentially identical in control conditions and under CGP55845 (*P* < 0.05; same cells). The difference between the average control pre-ACh mean current and the average Ih associated with the ***p***LTPextra provides a measure of the tonic GABA current (see Materials and Methods and Dominguez et al., 2014). Therefore, we tested for changes in ***t***GABA**_A_** induced by the ACh+depolarization protocol. The mean Ih had negative values of -78 ± 3 pA at -75 mV in control conditions and changed to -102 ± 7 pA with the IPSC potentiation, indicating a negative Ih shift that was 136% of the control ***t***GABA**_A_** values (*P* < 0.01; *N* = 5; **Figure 2A**, bottom traces and **Figures 2B,C**).

The mean peak amplitude of the spontaneous IPSCs (**_S_**IPSCs) increased from 22 ± 8 mV to 37 ± 3 pA following ACh, indicating an increase that was 168% of the control (*P* < 0.05; *N* = 5; **Figure 2A**, bottom traces and **Figures 2B–D,G**). In contrast, the mean **_S_**IPSCs frequency did not change after the ACh pulse (*P* > 0.05; **Figure 2A**, bottom traces and **Figures 2B,C,E,G**). These effects agree with the post-synaptic nature of the effects of ACh+depolarization. P_i_TX inhibited ***t***GABA**_A_** and the **_S_**IPSCs activity, implying that GABA**_A_**Rs mediated both tonic and synaptic currents (**Figure 2A**, bottom traces).

### There Were No Changes in IPSCs Following GABA Puffs

**FIGURE 3:**
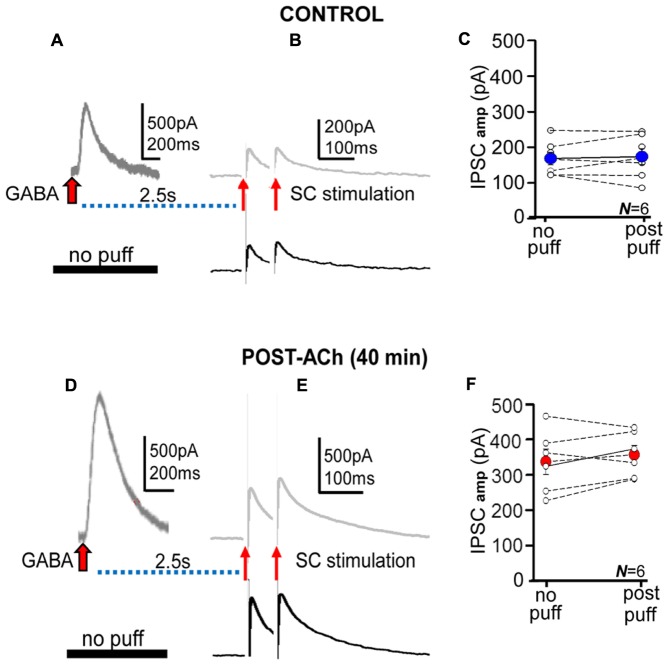
**The GABA puffs did not modify IPSCs. (A,B)** Representative records of control averaged ***p***GABA_A_ and IPSC pair evoked at delays of 2.5 s (upper) and of IPSC pair in the absence of the GABA puff (lower). **(C)** Scatter plot showing pooled data where small circles represent individual averaged responses (*N* = 6) and the large blue circle the corresponding mean in the absence (no puff) and following the GABA puff (post-puff). **(D–F)** Same as **(A–C)**, but Post-ACh (40 min). Note that the presence of the ACh puff and ***p***GABA_A_ does not modify control nor potentiated IPSCs.

The parallel increase in ***p***GABA**_A_, *t***GABA**_A_**, and IPSCs could suggest that an increased “ambient” GABA resulting from “spillover” and the puffed GABA caused the enhancement of currents and IPSPs. An increased number of GABA_A_Rs could take place in synaptic and also possibly in extrasynaptic sites, thus contributing to the result of ACh+depolarization. Therefore, we performed experiments in the same PCs in which IPSCs were evoked both in isolation and following GABA-puffs at delays of 2.5 s and in control and post-ACh (≈40 min) conditions (**Figure 3**). Both paired and isolated synaptic stimulation was at 0.1 s. In control pre-ACh conditions IPSC amplitudes were essentially identical when preceded or not by GABA puffs (**Figures 3A,B**). In addition, there were no statistically significant differences between both groups when data from different experiments was pooled (168.6 ± 17 pA pre-ACh and 173.7 ± 22 pA post-ACh, respectively, *P* > 0.05; *N* = 6; **Figure 3C**). In post-ACh conditions (≈40 min) IPSC amplitudes were larger but were not modified by the GABA puffs (**Figures 3D,E**), and there were no statistically significant differences between both groups when data from different experiments was pooled (336.7 ± 30 pA pre- and 353.4 ± 21 pA post-ACh, respectively; *P* > 0.05; *N* = 6; **Figure 3F**).

The absence of detectable interactions between ***p***GABA_A_ and IPSCs, suggests that two different receptor pools (i.e., extrasynaptic and synaptic) were activated by puffed and released GABA. These results could also suggest that the long-term enhancement of tonic and phasic inhibition shared key mechanisms and that GABA “spillover” did not play an dominant role in the effects of ACh+depolarization on IPSCs.

### Following the ACh Pulse *p*GABA_A_ Rapidly Increased during Depolarizing Steps

**FIGURE 4:**
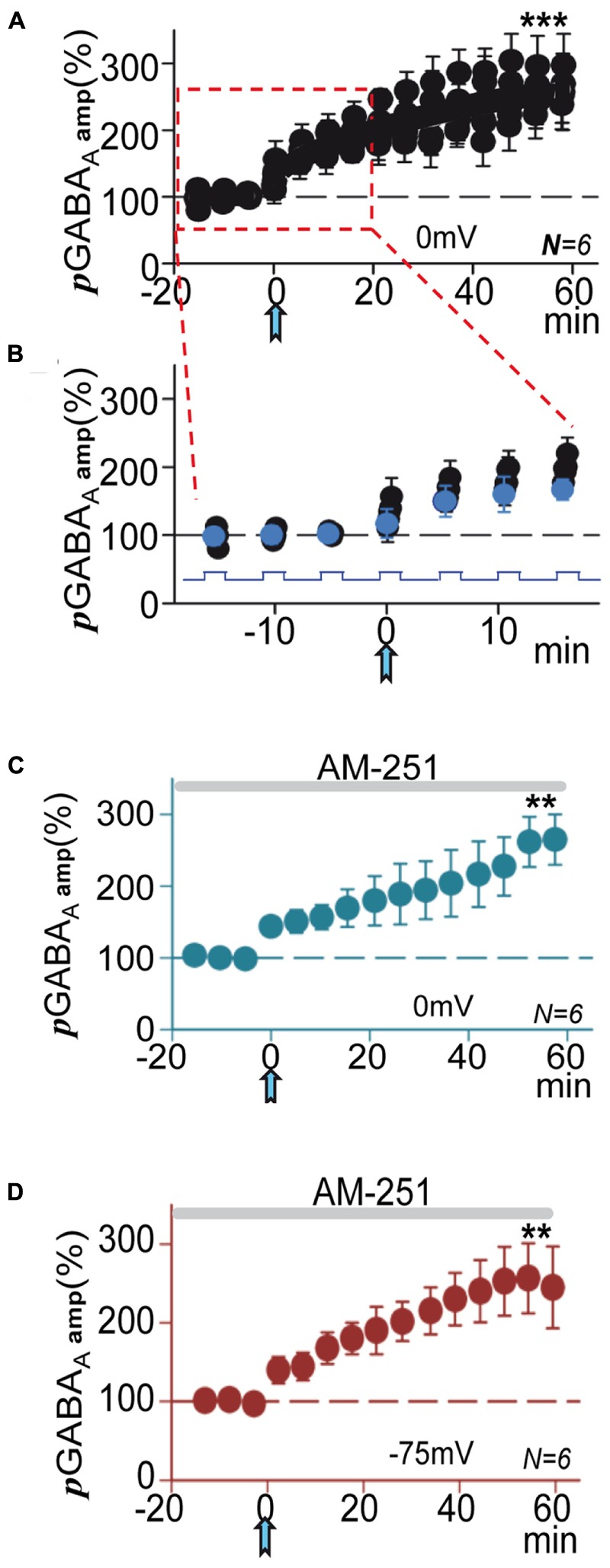
**Following the ACh pulse pGABA_A_ rapidly increased during depolarizing steps. (A)** Amplitude versus time plot where each data point represents the peak amplitude of ***p***GABA_A_s during all successive 0 mV steps, expressed as the proportion (%) of the mean value of first puff evoked currents recorded at 0 mV (see Materials and Methods and Dominguez et al., 2015) before and after the ACh pulse. Note that pre-ACh ***p***GABA_A_ did not change during steps, but post-ACh there were rapid enhancements of ***p***GABA_A_ during the 0 mV steps that lead to ***p***LTPextra. **(B)** Time expanded version taken from **(A)** (rectangle); the blue circles represent the first of the ***p***GABA_A_s evoked during the steps. **(C)** Average ***p***GABA_A_ peak amplitude versus time plot recorded at 0 mV under blockade of CB_1_Rs with AM-251. **(D)** Same as **(C)**, but recorded at -75 mV. **(C,D)** Were plotted as in **Figures 1D,E**. Asterisks indicate the significance level (^∗∗^*p* < 0.01, ^∗∗∗^*p* < 0.001).

We analyzed the temporal evolution of the peak amplitude of ***p***GABA_A_ at successive 0 mV steps (see Materials and Methods). Control pre-ACh ***p***GABA_A_s did not change during steps and were not enhanced by successive current steps. In contrast, following the ACh pulse there was a rapid enhancement of ***p***GABA_A_ during 0 mV steps that gradually increased in successive steps leading to a ***p***LTPextra (**Figures 4A,B**). Therefore, the potentiation process involved the rapid buildup with repeated depolarization of the machinery that gradually developed to finally stabilize with the potentiation.

### Endocannabinoids Did Not Contribute to *p*LTPextra

The activity of extrasynaptic GABA_A_Rs can also be enhanced by cannabinoids in a CB_1_R-independent manner (Golovko et al., 2015). Moreover, a robust hyperpolarization mediated by an increased K^+^ conductance, which can be blocked by the type 1 endocannabinoid receptor (CB_1_R) antagonist AM-251, has also been shown (Bacci et al., 2004). Therefore, we tested if ***p***LTPextra was modified by blockade of CB_1_R with AM-251 (2 μM). In these conditions ***p***LTPextra was essentially identical to that induced in control ACSF both at 0 and -75 mV (compare **Figures 4C,D** with **Figures 1D,E**), suggesting that endocannabinoids were not contributing to the effects of ACh+depolarization in our experimental conditions.

### The *p*GABA_A_ Decay Time Increased during the *p*LTPextra

**FIGURE 5:**
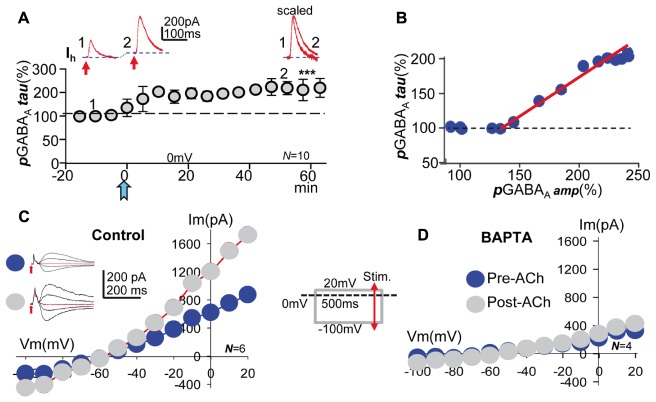
**Acetylcholine increased the decay time, the voltage- and GABA-sensitivity of pGABA_A_, effects that required a cytosolic Ca^2+^ raise.** (**A**, top) Representative record showing the smaller pre-ACh ***p***GABA_A_ taken at time 1 in (**A**, bottom). Larger ***p***GABA_A_, and outward change in mean Ih taken at time 2 in (**A**, bottom). (**A**, right) Same 1 and 2, but ***p***GABA_A_s scaled and superimposed. (**A**, bottom) Amplitude versus time plot where each data point represents ***tau*** values of ***p***GABA_A_ averaged over 5 min before and after the ACh pulse. **(B)** Plot of the functional relationship between peak ***p***GABA_A_ amplitude (taken from **Figure 2B**) and the ***tau p***GABA_A_ (taken from **A**, bottom), expressed as a proportion of the control pre-ACh ***tau***. (**C**, left) Representative pre- and post-ACh ***p***GABA_A_s recorded at -100, 0, 20, and 40 mV (arrows indicate GABA puffs). (**C**, right) Pre- and post-ACh I/V relationships (blue and gray circles, respectively) showing the linear voltage-dependence of ***p***GABA_A_ peak amplitude of the former and the increased average slope and outward rectification above ≈-40 mV of the latter, respectively. Note the unchanged reversal potential of control and potentiated ***p***GABA_A_. The inset show a simplified version of the I/V protocol. **(D)** Same as **(C)**, but under BAPTA-loading, showing that both pre- and post-ACh I/V relationships (blue and gray circles, respectively) tend to a similar linear model with small average slope. Asterisks indicate the significance level (^∗∗∗^*p* < 0.001).

We have shown that a increased decay time of IPSCs paralleled the synaptic GABA**_A_**-LTP (Dominguez et al., 2014), accordingly a increased decay time of ***p***GABA_A_ could also accompany ***p***LTPextra. The decay of ***p***GABA_A_ was well-fitted by a single exponential. The decay time (***tau***) of ***p***GABA_A_ gradually changed from the pre-ACh 84 ± 10 s to reach steady state values of 184 ± 9 s or a 219 ± 10% increase ≈40 min after ACh (*P* < 0.001; *N* = 10; **Figure 5A**). We plotted the peak ***p***GABA_A_ amplitude (taken from **Figure 2A**) versus ***p***GABA_A_***tau***, expressed as a proportion of the control pre-ACh ***p***GABA_A_ ***tau***. The plot revealed a linear correlation (*R* = 0.98; *N* = 10) between the mean peak amplitudes and ***tau*** of post-ACh ***p***GABA_A_ (**Figure 5B**). Therefore, ***p***LTPextra involved a gradual increase in the contribution of extrasynaptic GABA_A_Rs with a slower rate of desensitization than naïve receptors. Note also the outward shift in holding current following the ACh pulse (**Figure 5A**, insets 1 and 2).

### An Increased Contribution of Voltage-Sensitive GABA_A_Rs with Boosted GABA Sensitivity Underlies *p*LTPextra, Effects that Required a Cytosolic Ca^2+^ Rise

We have shown that an increased slope conductance and strong outward rectification of IPSCs typified the synaptic GABA**_A_**-LTP (Dominguez et al., 2014). Since GABA**_A_**-LTP and ***p***LTPextra share important properties, ***p***GABA_A_ could show an increased GABA- and a voltage-sensitivity. Therefore, we calculated I/V relationships of ***p***GABA_A_, which revealed that the control pre-ACh I/V relationship was linear with a small average slope (**Figure 5C**). In contrast, ≈40 min post-ACh the I/V plot showed an increased slope conductance and a strong outward rectification of ***p***GABA_A_ > -40 mV (**Figure 5C**). Importantly, the ACh challenge did not cause changes in the reversal potential of ***p***GABA_A_. We next tested BAPTA-loading (20 mM in the pipette solution), which blocked the increase in voltage- and GABA-sensitivity of ***p***GABA_A_ induced by ACh (**Figure 5D**). The above results taken together suggest that a Ca^2+^-induced increase in the contribution of slow desensitizing voltage-sensitive extrasynaptic GABA_A_Rs with boosted GABA affinity caused ***p***LTPextra as well as the synaptic GABA_A_-LTP (see Figure 6 in Dominguez et al., 2015).

**FIGURE 6:**
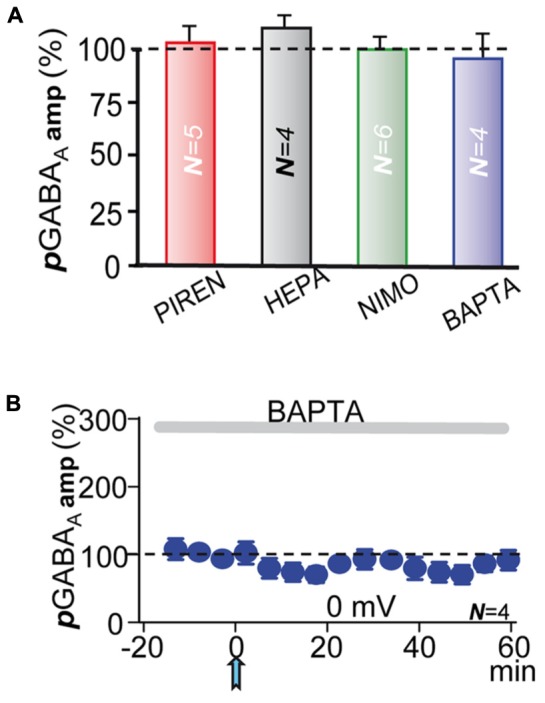
**M1-mAChRs and Ca^2+^ are required to induce the potentiation. (A)** Bar plot showing that blockade of M1-mAChRs by incubation with pirenzepine (**PIRE**, 1 μM), of Ca^2+^-release from IP_3_-sensitive stores by loading the PC with heparin (**HEPA**, 5 mg/ml), of L-type VGCC by incubation with nimodipine (**NIMO**, 10 μM), and intracellular Ca^2+^ chelation with **BAPTA** (20 μM in the pipette solution) prevented ***p***LTPextra. Average ***p***GABA_A_ peak amplitudes were calculated ≈40 min post-ACh. **(B) *p***GABA_A_ peak amplitude versus time plot showing the lack of ***p***LTPextra under BAPTA-loading. All recordings were at 0 mV.

### M1-mAChRs and Ca^2+^ Are Required to Induce the Potentiation

In CA1 pyramidal neurons depolarization coupled with M1-mAChR activation can induce a robust cytosolic Ca^2+^ signal, which can regulate inhibition through pre- and post-synaptic mechanisms (Dominguez et al., 2014, 2015). The ACh+depolarization protocol can increase intracellular Ca^2+^ both through Ca^2+^ release from IP_3_-sensitive intracellular stores and influx across L-type voltage-gated calcium channels (VGCCs) (Watanabe et al., 2006; Fernandez de Sevilla et al., 2008; Fernandez de Sevilla and Buno, 2010). Accordingly, we tested the effects of inhibiting Ca^2+^ release from IP_3_-sensitive stores by loading the PC with heparin (5 mg/ml in the pipette solution). Inhibition of IP_3_Rs prevented ***p***LTPextra and post-ACh ***p***GABA_A_ amplitudes reached values that were 118 ± 10% of the control (*P* > 0.05; *N* = 4; **Figure 6A**, HEPA). We also tested the effects of blocking L-type VGCC with nimodipine. Nimodipine (10 μM) inhibited ***p***LTPextra, stabilizing post-ACh ***p***GABA_A_ amplitudes at values that were 97 ± 6% of the control (*P* > 0.001; *N* = 6; **Figure 6A**, NIMO). Finally, we examined the effects of BAPTA-loading to inhibit the cytosolic Ca^2+^ rise. BAPTA-loading (20 mM in the pipette solution) blocked ***p***LTPextra and ***p***GABA_A_ amplitudes reached values that were 89 ± 5% of the control (*P* > 0.001; *N* = 5; **Figures 6A,B**, BAPTA). We also plotted the temporal evolution of ***p***GABA_A_ amplitudes under BAPTA-loading (**Figure 6B**).

### The Enhanced *t*GABA_A_ and IPSPs Reduced the Excitability of PCs

**FIGURE 7:**
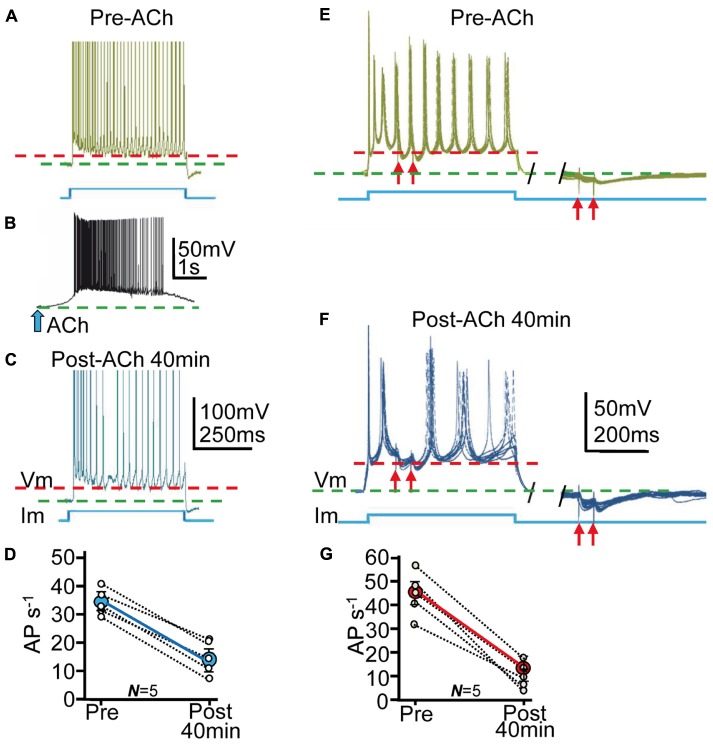
**The enhanced tGABA_A_ and IPSPs reduced the excitability of PCs. (A)** Representative example of current-clamp responses evoked by the depolarizing current pulse. The stimulation pulse is shown below (blue), and the red and green interrupted lines represent the AHP peaks and the resting potential, respectively (as in the rest of the figure). **(B)** Response evoked by ACh pulse. **(C)** Same as **(A)**, but ≈40 min post ACh. Note the decreased AP rate. **(D)** Plot where each data point (small open circles) represent the mean firing rate (AP/s) averaged over 5 min in Pre- and Post-ACh conditions (*N* = 5). The larger blue circle is the corresponding ensemble average showing the strong reduction of the AP rate induced ≈40 min after the ACh pulse. (**E**, left) Representative example of superimposed (7) current-clamp responses evoked by a repeated depolarizing current pulse protocol and paired pulse SC stimulation. (**E**, right) Same as left, but IPSP pair evoked during the return of the stimulation pulse. The red arrows represent stimulations. **(F)** Same as **(E)** but ≈40 min post-ACh. **(G)** Plot as in **(D)** showing changes in mean firing rate in Pre- and Post-ACh conditions (*N* = 5).

The tonic GABA current can play key roles in regulating network excitability (Bai et al., 2001; Semyanov et al., 2004), information processing (Mitchell and Silver, 2003; Chadderton et al., 2004) and behavior (Pavlov et al., 2009; Houston et al., 2012). Therefore, we performed current-clamp experiments to determine modifications in excitability of PCs induced by the enhanced ***t***GABA_A_ and IPSPs. We first depolarized PCs with 1 s duration current pulses applied every 10 s at twice the AP threshold during ≈10 min that triggered repetitive AP firing (**Figure 7A**). We interrupted the stimulation (≈3 min) and applied the ACh pulse that transiently depolarized the PC and evoked repetitive spiking (**Figure 7B**). Current pulse stimulation was resumed and the firing rate gradually decreased to stabilize ≈40 min later (**Figure 7C**), suggesting a gradual decrease in the excitability of the PCs. The ACh pulse induced a mean decay in firing rate from the control 35 ± 8 APs^-1^ to stabilize at 16 ± 9 APs^-1^ ≈40 min later (or a 48% decrease from the control; *P* < 0.05, *N* = 6; **Figure 7D**).

We next investigated the effects of both ***t***GABA_A_ and IPSCs on AP responses evoked by depolarizing current pulses. We depolarized PCs with 500 ms duration current pulses applied every 5–10 s at twice the AP threshold and simultaneously stimulated SCs to evoke pairs of IPSPs (see Materials and Methods; **Figures 7E,F**). We transiently interrupted the stimulation (≈3 min) and applied the ACh pulse that briefly depolarized the PC and evoked repetitive spiking (as above). Following the ACh pulse (≈40 min) there was a reduction in spike rate during depolarization from 45 ± 6 APs^-1^ to 18 ± 5 APs^-1^ (or a 39% decrease from control values; *P* < 0.01; *N* = 4; see Materials and Methods). The ACh pulse also increased IPSP amplitude (from 22 ± 11 to 48 ± 8 mV; *P* < 0.01; *N* = 4) or 211% of the control and delayed post-IPSP spikes from 22 ± 5 to 35 ± 10 ms (*P* < 0.01; *N* = 6; **Figures 7E–G**) or a 156% increase from control values.

An interpretation of the above results is that ACh+depolarization reduced the excitability of PCs through both an increased ***t***GABA**_A_** and IPSPs. The resting membrane potential (green interrupted lines in **Figure 7**) hyperpolarized by 8 ± 3 mV and the AP after-hyperpolarization (AHP) (red interrupted lines in **Figure 7**) increased 15 ± 8 mV during ≈40 min following the ACh pulse (*P* < 0.05; *N* = 10; **Figure 7**). The difference between the resting potential and the AHP provide a rough estimate of the depolarization attained during the current pulses, suggesting that more depolarization was required to reach AP threshold after the ACh+depolarization protocol.

## Discussion

Here, we analyzed the long-term effects of acetylcholine application paired with post-synaptic depolarization on both tonic and phasic GABA_A_ inhibition in CA1 PCs. Tonic inhibition results through activation by low concentrations of ambient GABA of slow desensitizing high-affinity voltage-sensitive extrasynaptic GABA_A_Rs (Kullmann, 2000; Semyanov et al., 2004; Glykys and Mody, 2007; Pavlov et al., 2009; Brickley and Mody, 2012). Tonic inhibition can be present in brain slices, where it can originate from synaptic GABA release, from reduced or reversed GABA uptake, and interestingly by non-synaptic GABA release (Bai et al., 2001; Semyanov et al., 2003; Petrini et al., 2004; Scimemi et al., 2005; Pavlov et al., 2009; Brickley and Mody, 2012). The ACh+depolarization protocol used here could increase ambient GABA both by spillover (Kullmann, 2000; Semyanov et al., 2004; Farrant and Nusser, 2005; Brickley and Mody, 2012; Kersante et al., 2013; Stephan and Friauf, 2014) and also by the GABA puffed.

Phasic inhibition follows from activation of low affinity synaptic GABA**_A_**Rs by brief release of high concentrations of GABA by exocytosis of presynaptic vesicles into the synaptic cleft (Farrant and Nusser, 2005). GABA_A_Rs mediating the two inhibitory modalities normally exhibit differences in subunit composition, GABA affinity and subcellular localization. However, ACh+depolarization induced a profound transformation that ended up with GABA_A_Rs displaying similar properties in extra- and synaptic compartments (Dominguez et al., 2014, 2015 and see Results). Accordingly, our results could suggest that the same intracellular mechanisms operate to increase the number of GABA_A_R of the same subtypes at synaptic and also possibly extrasynaptic sites. Importantly, GABA puffs increased ambient GABA, but did not modify IPSCs in control and potentiated conditions (**Figure 3**). In the controls there is a substantial difference in the GABA affinity of extra- and synaptic receptors (several orders of magnitude; Farrant and Nusser, 2005; Patel et al., 2016). Indeed, GABA increases to millimolar concentrations at the synaptic cleft to activate post-synaptic GABA_A_Rs, but only nanomolar concentrations are sufficient to activate extrasynaptic receptors during tonic inhibition (Santhakumar et al., 2006; Patel et al., 2016). Consequently, ambient GABA would readily activate extrasynaptic but not synaptic GABA_A_Rs because high ambient GABA concentrations, not usually attained *in vitro*, would be required to activate synaptic GABA_A_Rs.

In contrast, potentiated ***p***GABA_A_ and IPSCs display similar GABA affinity, outward rectification and decay kinetics (see Results and Dominguez et al., 2014, 2015), suggesting the presence of GABA_A_Rs with essentially identical biophysical properties and possibly similar subunit composition in both extra- and synaptic sites. However, although the increased ambient GABA activated extrasynaptic GABA_A_Rs, there was no detectable effect of GABA puffs on IPSCs. These results suggest that even with a significant increase in ambient GABA, the transmitter did not influence synaptic receptors during IPSCs in our experimental conditions.

The above results suggest that although GABA can flow in and out of the synaptic cleft the effects of outward GABA flow are clear-cut but those of inward flow are absent or unimportant. In contrast, when ambient GABA is significantly enhanced, such as high frequency stimulation of inhibitory inputs, increased interneuron activity, epileptic activity, and abnormal function of the GABA uptake, the massive increase in ambient GABA may modify synaptic responses (Barbour and Hausser, 1997; Farrant and Nusser, 2005; Glykys and Mody, 2007). However, the effects of an abnormally high concentration of GABA in the synaptic cleft can also reduce release through blockade of presynaptic Ca^2+^ channels *via* activation of presynaptic GABA_B_Rs (Wu and Saggau, 1997). Indeed, increasing ambient GABA by blocking neuronal GABA uptake can induce a strong GABA_B_R triggered presynaptic inhibition without signs of enhanced post-synaptic GABA_A_R activity (Dominguez et al., 2015).

Taken together our present and previous results (Dominguez et al., 2014, 2015), suggest that following the ACh pulse both an increased ambient GABA and number of slow desensitizing high-affinity voltage-sensitive GABA_A_Rs can occur. The increase in GABA_A_R number is likely to occur through the rapid lateral transit and clustering leading to enhanced responses (Kittler and Moss, 2003; Semyanov et al., 2004; Bannai et al., 2009; Pavlov et al., 2009; Ransom et al., 2010; Luscher et al., 2011; Brickley and Mody, 2012; Ransom et al., 2013; Dominguez et al., 2014, 2015). Interestingly, the dynamic lateral mobility of GABA_A_Rs can be enhanced by neuronal hyperactivity and operate in the 10s-of-milliseconds time range (Bannai et al., 2009; Dominguez et al., 2015), thus providing an exceptionally rapid negative feedback through the control of GABA_A_R number (Gaiarsa et al., 2002; Petrini et al., 2004; Luscher et al., 2011).

The ACh+depolarization protocol can trigger vigorous Ca^2+^ signals because the M1-mAChR-mediated blockade of K^+^ conductance raises the membrane resistance making the PC electrically compact (Benardo and Prince, 1982), boosting the depolarization-induced Ca^2+^ influx through L-type VGCC. In addition, activation of M1-mAChRs can induce Ca^2+^ release from IP_3_-sensitive stores (Watanabe et al., 2006; Fernandez de Sevilla and Buno, 2010). The strong cytosolic Ca^2+^ signal can trigger a rapid increase in the number of GABA_A_Rs at the membrane, which is critically dependent on Ca^2+^ influx through L-type VGCCs (Saliba et al., 2012).

Puffed and ambient GABA could activate different extrasynaptic GABA_A_Rs composed of α1/4/6β, α5- and δ-GABA**_A_**Rs subtypes (Caraiscos et al., 2004; Belelli et al., 2009; Brickley and Mody, 2012). CA1 PC synapses do not hold δ-GABA**_A_**Rs and α1/4/6β and α5- receptors are scarce at those synapses. However, lateral diffusion of extrasynaptic receptors can increase the number of α1/4/6β and α5- receptors at synapses (Semyanov et al., 2004; Bannai et al., 2009; Pavlov et al., 2009; Ransom et al., 2010, 2013; Brickley and Mody, 2012). α5- receptors show slow desensitization and outward rectification and high GABA affinity contributing to the tonic GABA current (Caraiscos et al., 2004). We have shown that these receptors contribute to the synaptic enhancement induced by ACh+depolarization (Dominguez et al., 2014, 2015), and could also function in the present experiments.

We cannot rule out a contribution of cholinergic-evoked astrocyte signaling, which plays important roles in balancing excitatory and inhibitory signals in the brain (Kang et al., 1998; Heja et al., 2012; Navarrete et al., 2012; Kersante et al., 2013; Stephan and Friauf, 2014). Astrocytes could control circuit operation in CA1 through glial GABA transporters (Kersante et al., 2013; Stephan and Friauf, 2014) and Ca^2+^ homeostasis (Araque et al., 2002). However, a direct demonstration of the possible signaling cascades that rule the effects of ACh+depolarization remain to be established.

It has been shown that the activity of extrasynaptic GABA_A_Rs can also be enhanced by cannabinoids in a CB_1_R-independent manner in neocortical pyramidal neurons (Golovko et al., 2015). Moreover, a prolonged CB_1_R-dependenet hyperpolarization mediated by an increased K^+^ conductance has been demonstrated in neocortical inhibitory interneurons (Bacci et al., 2004). These unconventional effects mediated by the release of endogenous cannabinoids, which could regulate synaptic strength and excitability, were not functional in our experimental conditions.

Changes in the Cl**^-^** concentration gradient caused by Cl**^-^** flux through activated GABA_A_Rs may globally modify GABA_A_-mediated activity (Woodin et al., 2003; Raimondo et al., 2012). However, the GABA_A_-LTP, which was induced in essentially identical experimental conditions and shares key mechanisms with the ***p***LTPextra and the increase ***t***GABA**_A_**, was unaffected by the Cl^-^ driving force, the Cl^-^ concentration gradient and K^+^ conductance block (Dominguez et al., 2014). In addition, the reversal potential of ***p***GABA_A_ did not change in the present conditions, suggesting that the effects of ACh+depolarization do not involve changes in the Cl^-^ concentration gradient.

We show that ***p***GABA_A_ inhibition displays both an increased slope conductance and a strong outward rectification (Dominguez et al., 2014) and thus exerts a stronger inhibition on excitatory inputs that depolarize the PC close to AP threshold, while it barely affects subthreshold inputs (Pavlov et al., 2009). Moreover, both the slope conductance and the rectification increase in function of time and the degree of PC activation (Dominguez et al., 2014), suggesting an homeostatic feedback role in the control of excitability (Mody, 2005). These effects could have a strong influence on network operation by maintaining the activity of the network within functional limits and could be a target for the treatment of hyperexcitable states.

## Author Contributions

SD, DF, and WB implemented conception and design of research; SD performed experiments; SD, DF, and WB analyzed data and interpreted results; SD, DF, and WB prepared figures; DF and WB wrote manuscript; SD, DF, and WB edited, revised, and approved final version of manuscript.

## Conflict of Interest Statement

The authors declare that the research was conducted in the absence of any commercial or financial relationships that could be construed as a potential conflict of interest.

## References

[B1] Alger B. E. (2002). Retrograde signaling in the regulation of synaptic transmission: focus on endocannabinoids. *Prog. Neurobiol.* 68 247–286. 10.1016/S0301-0082(02)00080-1 12498988

[B2] Araque A. Martin E. D. Perea G. Arellano J. I. Buno W. (2002). Synaptically released acetylcholine evokes Ca2+ elevations in astrocytes in hippocampal slices. *J. Neurosci.* 22 2443–2450. 11923408 10.1523/JNEUROSCI.22-07-02443.2002 PMC6758296

[B3] Bacci A. Huguenard J. R. Prince D. A. (2004). Long-lasting self-inhibition of neocortical interneurons mediated by endocannabinoids. *Nature* 431 312–316. 10.1038/nature02913 15372034

[B4] Bai D. Zhu G. Pennefather P. Jackson M. F. MacDonald J. F. Orser B. A. (2001). Distinct functional and pharmacological properties of tonic and quantal inhibitory postsynaptic currents mediated by gamma-aminobutyric acid(A) receptors in hippocampal neurons. *Mol. Pharmacol.* 59 814–824. 11259626 10.1124/mol.59.4.814

[B5] Bannai H. Levi S. Schweizer C. Inoue T. Launey T. Racine V. (2009). Activity-dependent tuning of inhibitory neurotransmission based on GABAAR diffusion dynamics. *Neuron* 62 670–682. 10.1016/j.neuron.2009.04.023 19524526

[B6] Barbour B. Hausser M. (1997). Intersynaptic diffusion of neurotransmitter. *Trends Neurosci.* 20 377–384. 9292962 10.1016/s0166-2236(96)20050-5

[B7] Belelli D. Harrison N. L. Maguire J. Macdonald R. L. Walker M. C. Cope D. W. (2009). Extrasynaptic GABAA receptors: form, pharmacology, and function. *J. Neurosci.* 29 12757–12763. 10.1523/JNEUROSCI.3340-09.2009 19828786 PMC2784229

[B8] Benardo L. S. Prince D. A. (1982). Ionic mechanisms of cholinergic excitation in mammalian hippocampal pyramidal cells. *Brain Res.* 249 333–344. 10.1016/0006-8993(82)90067-1 6291716

[B9] Brickley S. G. Mody I. (2012). Extrasynaptic GABA(A) receptors: their function in the CNS and implications for disease. *Neuron* 73 23–34. 10.1016/j.neuron.2011.12.012 22243744 PMC3399243

[B10] Caraiscos V. B. Elliott E. M. You-Ten K. E. Cheng V. Y. Belelli D. Newell J. G. (2004). Tonic inhibition in mouse hippocampal CA1 pyramidal neurons is mediated by alpha5 subunit-containing gamma-aminobutyric acid type A receptors. *Proc. Natl. Acad. Sci. U.S.A.* 101 3662–3667. 10.1073/pnas.0307231101 14993607 PMC373519

[B11] Castillo P. E. Chiu C. Q. Carroll R. C. (2011). Long-term plasticity at inhibitory synapses. *Curr. Opin. Neurobiol.* 21 328–338. 10.1016/j.conb.2011.01.006 21334194 PMC3092861

[B12] Chadderton P. Margrie T. W. Hausser M. (2004). Integration of quanta in cerebellar granule cells during sensory processing. *Nature* 428 856–860. 10.1038/nature02442 15103377

[B13] Connelly W. M. Errington A. C. Di Giovanni G. Crunelli V. (2013). Metabotropic regulation of extrasynaptic GABAA receptors. *Front. Neural Circuits* 7:171 10.3389/fncir.2013.00171 PMC3829460 24298239

[B14] Dennis S. H. Pasqui F. Colvin E. M. Sanger H. Mogg A. J. Felder C. C. (2015). Activation of muscarinic M1 acetylcholine receptors induces long-term potentiation in the hippocampus. *Cereb. Cortex* 26 414–426. 10.1093/cercor/bhv227 26472558 PMC4677984

[B15] Dominguez S. Fernandez de Sevilla D. Buno W. (2014). Postsynaptic activity reverses the sign of the acetylcholine-induced long-term plasticity of GABAA inhibition. *Proc. Natl. Acad. Sci. U.S.A.* 111 E2741–E2750. 10.1073/pnas.1321777111 24938789 PMC4084432

[B16] Dominguez S. Fernandez de Sevilla D. Buno W. (2015). Acetylcholine facilitates a depolarization-induced enhancement of inhibition in rat CA1 pyramidal neurons. *Cereb. Cortex* 10.1093/cercor/bhv276 [Epub ahead of print]. 26620268

[B17] Farrant M. Nusser Z. (2005). Variations on an inhibitory theme: phasic and tonic activation of GABA(A) receptors. *Nat. Rev. Neurosci.* 6 215–229. 10.1038/nrn1625 15738957

[B18] Fernandez de Sevilla D. Buno W. (2010). The muscarinic long-term enhancement of NMDA and AMPA receptor-mediated transmission at Schaffer collateral synapses develop through different intracellular mechanisms. *J. Neurosci.* 30 11032–11042. 10.1523/JNEUROSCI.1848-10.2010 20720110 PMC6633467

[B19] Fernandez de Sevilla D. Nunez A. Borde M. Malinow R. Buno W. (2008). Cholinergic-mediated IP3-receptor activation induces long-lasting synaptic enhancement in CA1 pyramidal neurons. *J. Neurosci.* 28 1469–1478. 10.1523/JNEUROSCI.2723-07.2008 18256268 PMC6671582

[B20] Gaiarsa J. L. Caillard O. Ben-Ari Y. (2002). Long-term plasticity at GABAergic and glycinergic synapses: mechanisms and functional significance. *Trends Neurosci.* 25 564–570. 10.1016/S0166-2236(02)02269-5 12392931

[B21] Glykys J. Mody I. (2007). The main source of ambient GABA responsible for tonic inhibition in the mouse hippocampus. *J. Physiol.* 582(Pt. 3) 1163–1178. 10.1113/jphysiol.2007.134460 17525114 PMC2075237

[B22] Golovko T. Min R. Lozovaya N. Falconer C. Yatsenko N. Tsintsadze T. (2015). Control of inhibition by the direct action of cannabinoids on GABAA receptors. *Cereb. Cortex* 25 2440–2455. 10.1093/cercor/bhu045 24646614

[B23] Hasselmo M. E. (2006). The role of acetylcholine in learning and memory. *Curr. Opin. Neurobiol.* 16 710–715. 10.1016/j.conb.2006.09.002 17011181 PMC2659740

[B24] Heja L. Nyitrai G. Kekesi O. Dobolyi A. Szabo P. Fiath R. (2012). Astrocytes convert network excitation to tonic inhibition of neurons. *BMC Biol.* 10:26 10.1186/1741-7007-10-26 PMC3342137 22420899

[B25] Houston C. M. McGee T. P. Mackenzie G. Troyano-Cuturi K. Rodriguez P. M. Kutsarova E. (2012). Are extrasynaptic GABAA receptors important targets for sedative/hypnotic drugs? *J. Neurosci.* 32 3887–3897. 10.1523/JNEUROSCI.5406-11.2012 22423109 PMC4620914

[B26] Kang J. Jiang L. Goldman S. A. Nedergaard M. (1998). Astrocyte-mediated potentiation of inhibitory synaptic transmission. *Nat. Neurosci.* 1 683–692. 10.1038/3684 10196584

[B27] Kano M. Ohno-Shosaku T. Hashimotodani Y. Uchigashima M. Watanabe M. (2009). Endocannabinoid-mediated control of synaptic transmission. *Physiol. Rev.* 89 309–380. 10.1152/physrev.00019.2008 19126760

[B28] Kersante F. Rowley S. C. Pavlov I. Gutierrez-Mecinas M. Semyanov A. Reul J. M. (2013). A functional role for both -aminobutyric acid (GABA) transporter-1 and GABA transporter-3 in the modulation of extracellular GABA and GABAergic tonic conductances in the rat hippocampus. *J. Physiol.* 591(Pt. 10) 2429–2441. 10.1113/jphysiol.2012.246298 23381899 PMC3678035

[B29] Kittler J. T. Moss S. J. (2003). Modulation of GABAA receptor activity by phosphorylation and receptor trafficking: implications for the efficacy of synaptic inhibition. *Curr. Opin. Neurobiol.* 13 341–347. 10.1016/S0959-4388(03)00064-3 12850219

[B30] Kullmann D. M. (2000). Spillover and synaptic cross talk mediated by glutamate and GABA in the mammalian brain. *Prog. Brain Res.* 125 339–351. 10.1016/S0079-6123(00)25023-1 11098670

[B31] Luscher B. Fuchs T. Kilpatrick C. L. (2011). GABAA receptor trafficking-mediated plasticity of inhibitory synapses. *Neuron* 70 385–409. 10.1016/j.neuron.2011.03.024 21555068 PMC3093971

[B32] Markram H. Segal M. (1990). Long-lasting facilitation of excitatory postsynaptic potentials in the rat hippocampus by acetylcholine. *J. Physiol.* 427 381–393. 10.1113/jphysiol.1990.sp018177 2145426 PMC1189936

[B33] Mitchell S. J. Silver R. A. (2003). Shunting inhibition modulates neuronal gain during synaptic excitation. *Neuron* 38 433–445. 10.1016/S0896-6273(03)00200-9 12741990

[B34] Mody I. (2005). Aspects of the homeostaic plasticity of GABAA receptor-mediated inhibition. *J. Physiol.* 562(Pt. 1) 37–46. 10.1113/jphysiol.2004.077362 15528237 PMC1665492

[B35] Navarrete M. Perea G. Fernandez de Sevilla D. Gomez-Gonzalo M. Nunez A. Martin E. D. (2012). Astrocytes mediate in vivo cholinergic-induced synaptic plasticity. *PLoS Biol.* 10:e1001259 10.1371/journal.pbio.1001259 PMC3279365 22347811

[B36] Nurse S. Lacaille J. C. (1999). Late maturation of GABA(B) synaptic transmission in area CA1 of the rat hippocampus. *Neuropharmacology* 38 1733–1742. 10.1016/S0028-3908(99)00122-7 10587089

[B37] Patel B. Bright D. P. Mortensen M. Frolund B. Smart T. G. (2016). Context-dependent modulation of GABAAR-mediated tonic currents. *J. Neurosci.* 36 607–621. 10.1523/JNEUROSCI.2047-15.2016 26758848 PMC4710777

[B38] Pavlov I. Savtchenko L. P. Kullmann D. M. Semyanov A. Walker M. C. (2009). Outwardly rectifying tonically active GABAA receptors in pyramidal cells modulate neuronal offset, not gain. *J. Neurosci.* 29 15341–15350. 10.1523/JNEUROSCI.2747-09.2009 19955387 PMC6665960

[B39] Petrini E. M. Marchionni I. Zacchi P. Sieghart W. Cherubini E. (2004). Clustering of extrasynaptic GABA(A) receptors modulates tonic inhibition in cultured hippocampal neurons. *J. Biol. Chem.* 279 45833–45843. 10.1074/jbc.M407229200 15317810

[B40] Raimondo J. V. Markram H. Akerman C. J. (2012). Short-term ionic plasticity at GABAergic synapses. *Front. Synaptic Neurosci.* 4:5 10.3389/fnsyn.2012.00005 PMC3472547 23087642

[B41] Ransom C. B. Tao W. Wu Y. Spain W. J. Richerson G. B. (2013). Rapid regulation of tonic GABA currents in cultured rat hippocampal neurons. *J. Neurophysiol.* 109 803–812. 10.1152/jn.00460.2012 23114210 PMC3567397

[B42] Ransom C. B. Wu Y. Richerson G. B. (2010). Postdepolarization potentiation of GABAA receptors: a novel mechanism regulating tonic conductance in hippocampal neurons. *J. Neurosci.* 30 7672–7684. 10.1523/JNEUROSCI.0290-10.2010 20519542 PMC2902370

[B43] Robinson L. Platt B. Riedel G. (2011). Involvement of the cholinergic system in conditioning and perceptual memory. *Behav. Brain Res.* 221 443–465. 10.1016/j.bbr.2011.01.055 21315109

[B44] Saliba R. S. Kretschmannova K. Moss S. J. (2012). Activity-dependent phosphorylation of GABAA receptors regulates receptor insertion and tonic current. *EMBO J.* 31 2937–2951. 10.1038/emboj.2012.109 22531784 PMC3395084

[B45] Santhakumar V. Hanchar H. J. Wallner M. Olsen R. W. Otis T. S. (2006). Contributions of the GABAA receptor alpha6 subunit to phasic and tonic inhibition revealed by a naturally occurring polymorphism in the alpha6 gene. *J. Neurosci.* 26 3357–3364. 10.1523/JNEUROSCI.4799-05.2006 16554486 PMC2247415

[B46] Scimemi A. Semyanov A. Sperk G. Kullmann D. M. Walker M. C. (2005). Multiple and plastic receptors mediate tonic GABAA receptor currents in the hippocampus. *J. Neurosci.* 25 10016–10024. 10.1523/JNEUROSCI.2520-05.2005 16251450 PMC6725560

[B47] Semyanov A. Walker M. C. Kullmann D. M. (2003). GABA uptake regulates cortical excitability via cell type-specific tonic inhibition. *Nat. Neurosci.* 6 484–490. 10.1038/nn1043 12679782

[B48] Semyanov A. Walker M. C. Kullmann D. M. Silver R. A. (2004). Tonically active GABA A receptors: modulating gain and maintaining the tone. *Trends Neurosci.* 27 262–269. 10.1016/j.tins.2004.03.005 15111008

[B49] Stephan J. Friauf E. (2014). Functional analysis of the inhibitory neurotransmitter transporters GlyT1, GAT-1, and GAT-3 in astrocytes of the lateral superior olive. *Glia* 62 1992–2003. 10.1002/glia.22720 25103283

[B50] Watanabe S. Hong M. Lasser-Ross N. Ross W. N. (2006). Modulation of calcium wave propagation in the dendrites and to the soma of rat hippocampal pyramidal neurons. *J. Physiol.* 575(Pt. 2) 455–468. 10.1113/jphysiol.2006.114231 16809362 PMC1819440

[B51] Woodin M. A. Ganguly K. Poo M. M. (2003). Coincident pre- and postsynaptic activity modifies GABAergic synapses by postsynaptic changes in Cl- transporter activity. *Neuron* 39 807–820. 10.1016/S0896-6273(03)00507-5 12948447

[B52] Wu L. G. Saggau P. (1997). Presynaptic inhibition of elicited neurotransmitter release. *Trends Neurosci.* 20 204–212. 10.1016/S0166-2236(96)01015-6 9141196

